# Adult hookworms (*Necator* spp.) collected from researchers working with wild western lowland gorillas

**DOI:** 10.1186/s13071-016-1357-0

**Published:** 2016-02-09

**Authors:** Barbora Kalousová, Hideo Hasegawa, Klára J. Petrželková, Tetsuya Sakamaki, Takanori Kooriyma, David Modrý

**Affiliations:** Department of Pathology and Parasitology, Faculty of Veterinary Medicine, University of Veterinary and Pharmaceutical Sciences, Palackeho tr. 1946/1, 612 42 Brno, Czech Republic; Department of Biology, Faculty of Medicine, Oita University, Hasama, Yufu, Oita 879-5593 Japan; Institute of Vertebrate Biology, Academy of Sciences of the Czech Republic, Kvetna 8, 603 65 Brno, Czech Republic; Biology Centre, Institute of Parasitology, Academy of Sciences of the Czech Republic, Branisovska 31, 370 05 Ceske Budejovice, Czech Republic; Liberec Zoo, Lidove sady 425/1, 460 01 Liberec, Czech Republic; Primate Research Institute, Kyoto University, Inuyama, Aichi 484-8506 Japan; Department of Veterinary Science, School of Veterinary Medicine, Rakuno Gakuen University, 582 Bunkyodai-Midori, Ebetsu, Hokkaido 069-8501 Japan; Central European Institute for Technology (CEITEC), University of Veterinary and Pharmaceutical Sciences, Palackeho 1946/1, 612 42 Brno, Czech Republic

**Keywords:** *Necator* spp, *Necator gorillae*, *Necator americanus*, Hookworm, Morphology, Human infection

## Abstract

**Background:**

In general, studies on the diversity of strongylid nematodes in endangered host species are complicated as material obtained by non-invasive sampling methods has limited value for generic and species identification. While egg morphology barely allows assignment to family, the morphology of cultivated infective third stage larvae provides a better resolution at the generic level but cannot be used for exact species identification. Morphology-based taxonomic approaches greatly depend on the examination of adult worms that are usually not available.

**Methods:**

Hookworm parasites in two European researchers, who participated in gorilla research in the Central African Republic, were expelled after anthelmintic treatment to the faeces, collected and morphologically examined. A male worm discharged naturally from a wild bonobo (*Pan paniscus*) in Congo was also examined for comparison.

**Results:**

Two species of *Necator* were identified in researchers’ faecal material: *Necator americanus* (Stiles, 1902) and *N. gorillae* Noda & Yamada, 1964; the latter species differed in having a smaller body, smaller buccal cavity and shorter spicules with spade-shaped membranes situated distally. Males of *N. gorillae* also possessed unusual cuticular thickenings on the dorsal side of the prebursal region of the body. These characters, shared with the male worm from the bonobo, correspond well to the description of *N. gorillae* described from gorillas in Congo.

**Conclusions:**

Based on the morphology of the hookworms recovered in this study and previous molecular analyses of larvae developed from both humans and western lowland gorillas (*Gorilla gorilla gorilla*) from this locality, we conclude that the researchers became infected with gorilla hookworms during their stay in the field. This is the first report of infection with a *Necator* species other than *N. americanus* in humans.

## Background

Strongylid nematodes are an important component of helminth communities found in large herbivorous mammals [1, 2]. In general, studies on their diversity in endangered host species are complicated as material obtained by non-invasive sampling methods has limited value for generic and species identification. While egg morphology barely allows assignment to family, the morphology of cultivated infective third stage (L3) larvae provides a better resolution at the generic level [3] but cannot be used for exact species identification. Morphology-based taxonomic approaches greatly depend on the examination of adult worms, which are mostly obtained only during necropsies and thus are lacking. As a result DNA-based taxonomy suffers from the absence of comparative sequences from well-identified individuals.

Until recently, it was believed that *Necator americanus* (Stiles, [4] ) is the only species of *Necator* parasitic in humans [5]. However, Hasegawa et al. [6] recently proved by DNA sequence analysis from infective third stage larvae raised from faecal cultures that at least two *Necator* spp. are shared by humans, western lowland gorillas *Gorilla gorilla gorilla* Savage, and central chimpanzees *Pan troglodytes troglodytes* Blumenbach, in the tropical forest in Dzanga Sangha Protected Areas (DSPA), Central African Republic (CAR). The L3 larvae sequenced showed morphological characteristics of *Necator* spp.*,* having a distinct spear-like structure in the buccal cavity and clear transverse striations on the sheath [3]. However, it was impossible to assign the larvae to species based on their morphology. Based on the DNA sequence profile (ITS rDNA and *cox*1 mtDNA), one of the detected species was considered as typical *N. americanus*, while the taxonomic placement of the second taxon was impossible and was referred to as *Necator* sp.

After returning from the field survey of gorillas in DSPA (CAR), three European researchers were diagnosed with hookworm infections. DNA sequencing on the L3 larvae cultured from two of them suggested a mixed infection with *N. americanus* and other *Necator* spp. We attempted to collect the adult hookworms from their faeces after anthelmintic treatment. Here we present the morphology of the hookworms recovered as the first report of a species of *Necator* other than *N. americanus* from humans.

## Methods

All procedures performed in studies involving human participants were in accordance with the ethical standards of the institutional and/or national research committee (Ethical Commission of the Biology Centre of the Academy of Sciences of the Czech Republic) and with the 1964 Helsinki declaration and its later amendments or comparable ethical standards.

Two researchers studying western lowland gorillas in DSPA (CAR) were coproscopically diagnosed positive for strongylid nematodes. Both were treated by albendazole (400 mg, a single dose) and all expelled faeces during and 2 days after the treatment were collected and fixed in 4 % formaldehyde solution.

Researcher A: A 23-year-old woman who spent nine months in the field in DSPA from November 2010 to August 2011. Hookworm infection was diagnosed 11 months after returning from CAR.

Researcher B: A 37-year-old woman who has repeatedly participated in DSPA field research for 2–3 month periods in 2007–2012. She was diagnosed with hookworm infection in October 2012.

Fixed faeces were washed with running tap water on piled strainers with a mesh aperture size of 2.8 mm, 1.00 mm, 0.60 mm, 0.25 mm, and 0.106 mm, respectively. The remaining residue on each strainer was transferred to a glass dish, examined under a stereomicroscope for the presence of nematodes; the worms collected were preserved in 70 % ethanol. For light microscopy examination, the nematodes were cleared in glycerol-ethanol solution by evaporation of ethanol, and mounted on glass slides with 50 % glycerol aqueous solution, or cleared in lactophenol solution. The spicules were excised from one worm using a fine needle to observe its distal ends. All measurements are based on glycerol-mounted specimens and are presented in micrometres unless indicated otherwise. Drawings are made with the aid of a Nikon drawing tube attached to a Nikon Optiphot microscope equipped with interference contrast.

Comparative material examined: a single male adult of *Necator gorillae* from the bonobo *Pan paniscus* Schwarz from Wamba, Congo, fixed in 4 % formaldehyde solution; 20 male and 20 female *N. americanus* adult worms from a woman in Oita, Japan already identified [7].

The specimens are deposited at the Department of Pathology and Parasitology, University of Veterinary and Pharmaceutical Sciences, Brno (Czech Republic) under accession numbers VFUNK1, VFUNB1–VFUNB16.

## Results

Seventeen individuals of *Necator* spp. were recovered from the researcher A and a single worm was recovered from researcher B. These were identified as two species based on detailed morphological study as described below.

### Morphological descriptions

#### *Necator americanus* (Stiles, [4]) from a human

[Based on two males and nine females from researcher A and one male from researcher B.]

**Fig. 1:**
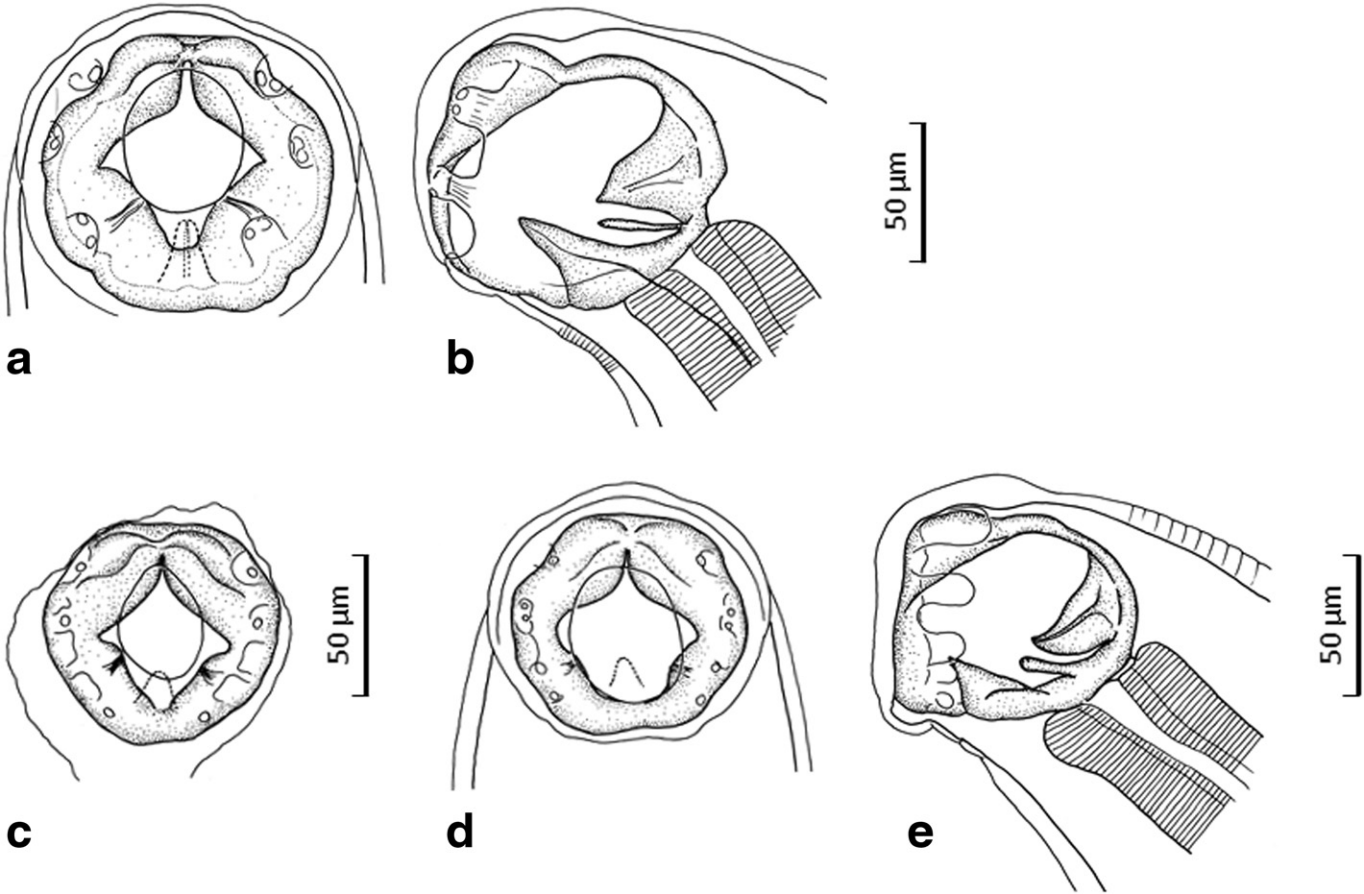
Cephalic end of *Necator americanus* (**a**, **b**) and *N. gorillae* (**c**–**e**). a, b, Male of *N. americanus* collected from a researcher, dorsal (**a**) and right lateral (**b**) views; c, Male of *N. gorillae* collected from a researcher, dorsal view; d, e Male of *N. gorillae* collected from a bonobo, dorsal (**d**) and right lateral (**e**) views

*General*. Anterior extremity strongly tilted dorsally; posterior body bent ventrally. Cuticle thick, with transverse striations. Oral aperture oval. Buccal capsule well developed. Buccal collar absent. Ventral cutting plates well developed, with slightly angular free edge corner; dorsal cutting plates present. Dorsal cone supported by two subventral plates (Fig. 1a, b); oesophagus club-shaped.

**Fig. 2:**
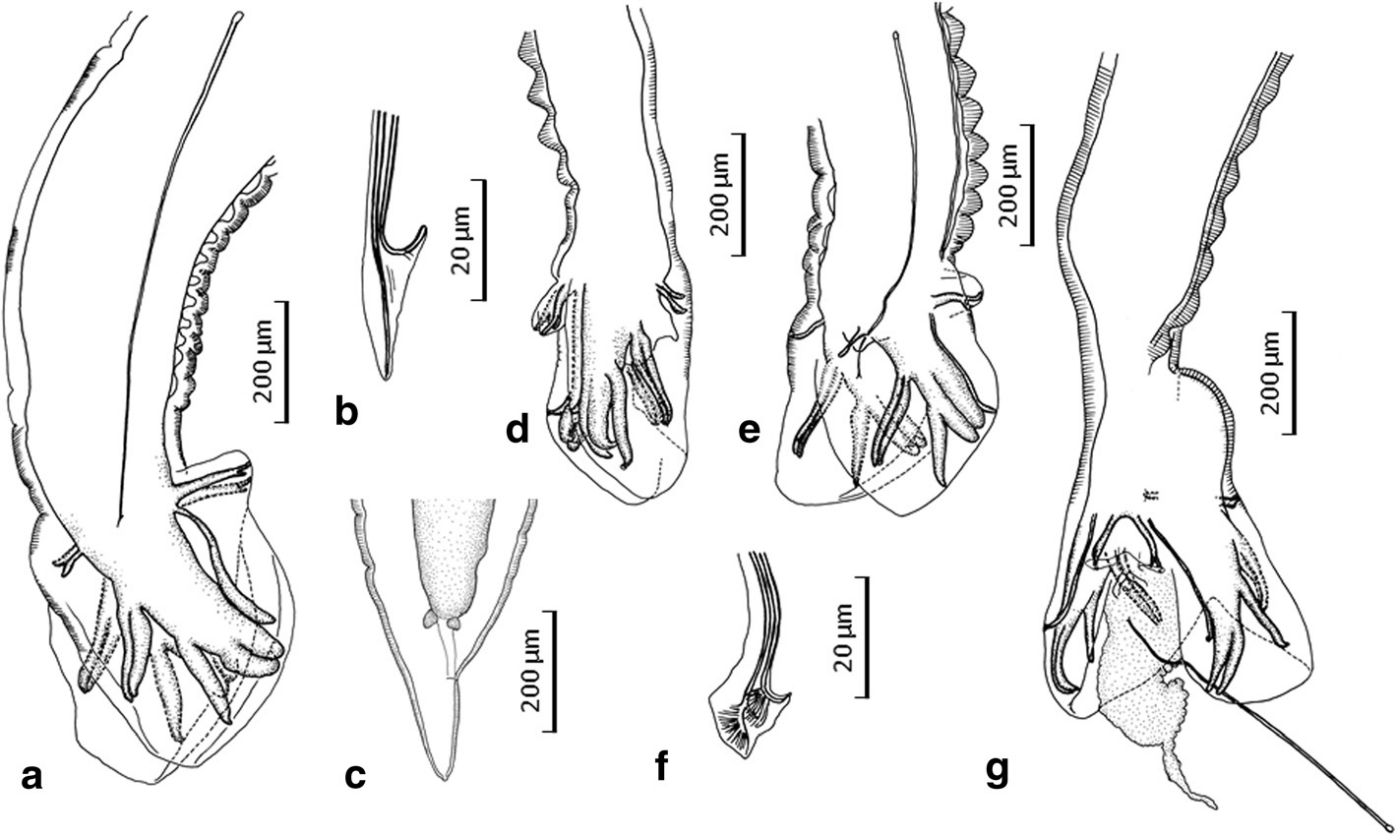
Posterior extremities and distal end of spicules of the hookworms studied. **a**, **b**, Male of *N. americanus* collected from a researcher, left lateral view (**a**) and its spicule end (**b**) (note wrinkled dorsal cuticle in the prebursal region); **c**, Female of *N. gorillae* collected from a researcher, left lateral view; d, Male of *N. gorillae* collected from a researcher, right lateral view (note cuticular thickenings in the dorsal prebursal region); **e**, **f**, Male of *N. gorillae* collected from a researcher, left lateral view (**e**) (note cuticular thickenings in the dorsal prebursal region) and spicule end excised (**f**); **g**, Male of *N. gorillae* collected from a bonobo, with body twisted at base of bursa and inside matter with spicules extruded from the posterior end; note cuticular thickenings in the dorsal prebursal region

*Male* (*n* = 3). Body length 7.07–8.54 mm; width 344–390 at mid-body. Buccal capsule 106–114 × 89–99. Oesophagus 679–698 long, 134–154 wide near posterior end (*n* = 3). Nerve-ring 334–425 (*n* = 2), deirids and excretory pore 425–439 (*n* = 2) and 397–410, respectively, from cephalic extremity. Spicules slender, 865–975 long (corresponding to 11.6–12.9 % of worm length) with fused distal ends; one spicule forming recurved barb, the other straight, forming a spear (Fig. 2a, b). Dorsal bursal rays much shorter than laterals, diverged from each other at base, and distally bifid into unequal offshoots (Fig. 2a).

*Female* (*n* = 9). Body length 7.03–14.2 mm; width 299–540 at mid-body. Buccal capsule 96–123 × 83–107. Oesophagus 594–830 long, 128–154 wide near posterior end. Nerve-ring 297–585, deirids and excretory pore 311–618 and 247–618, respectively, from cephalic extremity. Vulva 2.40–5.93 mm from cephalic extremity (corresponding to 33.1–46.1 % of body length). Tail conical, pointed, lacking terminal spike, 182–274 long.

#### *Necator gorillae* Noda & Yamada, [8] from a human

[Based on five males and two females from researcher A.].

*General*. Resembling *N. americanus* but smaller. Oral aperture oval. Ventral cutting plates well developed, with round free edge corner; dorsal cutting plates often overlapped by oral aperture rim (Fig. 1c).

*Male* (1 entire worm and 4 fragmented worms lacking anterior body). Body length 4.88 mm (*n* = 1); width 285–286 at mid-body. Buccal capsule 88 × 75 (Fig. 1c). Oesophagus 514 long, 70 wide near posterior end. Fragmented worms with 7–9 cuticular thickenings on dorsal side of prebursal portion (Fig. 2d, e); entire male with greatly wrinkled cuticle in posterior body, obscuring cuticular thickenings. Spicules slender, 489–566 long (corresponding to 10.1 % of worm length) (*n* = 1); one spicule recurved, the other straightened distally; small spade-shaped membrane with fine lines connecting both distal ends of spicules present (Fig. 2d, e). Bursal rays thin except lateral rays; ventral rays running together along whole length; externolateral rays diverged from mediolateral rays, widely separated distally; externodorsal rays very thin, attached to posterolateral rays along nearly entire length, but distally diverged; dorsal rays thin, diverged from each other at base, and divided distally into two unequal offshoots (Fig. 2d, e).

*Female* (*n* = 2). Body length 6.00–6.19 mm; width 293–390 at mid-body. Buccal capsule 98–99 × 84–88. Oesophagus 580–585 long, 138–141 wide near posterior end. Nerve-ring, deirids and excretory pore 288–354, 170–354 and 189–278, respectively, from cephalic extremity. Vulva 2.24 mm from cephalic extremity (corresponding to 37.3 % of body length) (*n* = 1). Tail conical, pointed, lacking terminal spike, 169–173 long (Fig. 2c) (morphology identical with that of *N. americanus*).

#### *Necator gorillae* Noda & Yamada, [8] from a bonobo

*Male* (*n* = 1). Morphology identical with *N. gorillae* from humans (see above). Body length 7.38 mm¸ width 312 at mid-body. Buccal capsule 88 × 77 (Fig. 1d, e). Oesophagus 598 long, 122 wide. Nerve-ring, deirids and excretory pore 321, 481 and 411, respectively, from cephalic extremity. Spicules 570 long (corresponding to 7.7 % of worm length). Seven transverse cuticular thickenings present on dorsal side of prebursal portion (Fig. 2g).

### Remarks

The worms identified as *N. americanus* in the present study were morphologically identical to *N. americanus* collected from a woman in Japan*,* including the cephalic structure and distal ends of the spicules [9, 10]. The presence of *N. americanus* in the same material from researcher B was previously proved by sequence analysis of DNA from L3 larvae raised by coproculture [6].

We compared the specimens of *N. gorillae* identified in our study with the species previously described in great apes [11, 12], i.e. *N. exilidens* Cummins, [13], *N. congolensis* Gedoelst, [12] and *N. gorillae* Noda & Yamada, [8]. *Necator exilidens* differs from the other two species in the shape of the mouth, the length of the spicules and the shape of the ventral cutting plates. *Necator exilidens* has spindle-shaped mouth whereas the mouth in the other two species has an ovoidal shape. The spicules of *N. exilidens* are also more than twice as long (1,360 μm) in comparison with those of *N. congolensis* and *N. gorillae* (both < 600 μm). All male specimens of *N. gorillae* recovered in our study possess spicules shorter than 600 μm with distal small spade-shaped membrane with fine lines. Ventral cutting plates of *N. exilidens* are round (*vs* angular in the other species). Because of all of these characteristics, the distinction of the present material from *N. exilidens* is apparent. The difference between *N. congolensis* and *N. gorillae* comprises the absence of dorsal cutting plates in *N. congolenses,* which are present in *N. gorillae* examined here. The most important aspect which distinguishes *N. gorillae* from other *Necator* spp. is the presence of prebursal dorsal cuticular thickenings; these ridges do not seem to be resulting from body constriction as the subcuticle layer showed no wrinkles. This is in sharp contrast with the almost smooth dorsal cuticle in *N. americanus* (Fig. 2a) and other previously described species [12]. The prebursal dorsal thickenings have been described only for *N. gorillae* collected from a wild western lowland gorila, which was caught in Congo, transported to Japan and died soon after arrival [8]. Additionally, the male hookworm from the comparative bonobo material shared morphological characteristics with *N. gorillae* (Figs. 1a, 2 g) and we assume these to be conspecific. Other morphological features and measurements of the male *N. gorillae* from the researcher and the bonobo also agree well with those of *N. gorillae*.

In the original description of *N. gorillae* by Noda & Yamada [8], the buccal capsule was given as 66–68 × 58–60 μm in males and 68–74 × 60–66 in females, i.e. much smaller than in the present worms identified as *N. gorillae*. However, the buccal cavity of the male shown in Fig. 2 in Noda & Yamada [8] measures 88 × 80 μm, i.e. almost equal to that in males of *N. gorillae* described here from humans and the bonobo. Finally, the morphology of the spicules of *N. gorillae*, as described by Noda & Yamada [8], corresponds to that observed by us in the worms identified as *N. gorillae*, regardless of some terminological inconsistencies.

## Discussion

The species composition of *Necator* spp. parasitising African great apes is complex and remains unclear. Four species, i.e. *Necator americanus*, *N. exilidens* (from a chimpanzee), *N. congolensis* (from chimpanzees in Congo) and *N. gorillae* (from western lowland gorilla in Congo), have been described [4, 8, 12, 13]. The key distinguishing features of the above-mentioned species are not clear, especially those of *N. exilidens*, *N. congolensis* and *N. gorillae*. As the type-material of these species is not available at the present time, direct comparison with our specimens was impossible. Nevertheless, the original description of *N. gorillae* outlined by Noda & Yamada [8] is detailed enough to allow thorough morphological comparison as presented above.

This study is a follow up to previous work by Hasegawa et al*.* [6], who described the DNA sequence profile (ITS and *cox*1) of L3 larvae raised from stools of researchers and confirmed that researchers (and great apes) at DSPA were infected with more than one species of *Necator*. Initially identified *Necator* spp. clustered in both rDNA and mtDNA trees with *N. americanus*, the most common human-infecting species of *Necator*; this corresponds well to the presence of adult *N. americanus* described in our study. The present specimens of *N. americanus* exhibited identical morphology with those expelled from the Japanese woman [7]. It is also noteworthy that *Necator* sp. with ITS2 sequence closely resembling the type III previously recorded from a human in DSPA [6], was recently found in bonobo from the Congo [14].

Other genotypes/haplotype groups, dominant among larvae obtained from gorillas and chimpanzees, but present also in humans, were suggested to belong to other previously described great ape hookworm taxa i.e., *N. congolensis*, *N. exilidens* or *N. gorillae* [6]. Based on the morhological data obtained and presented in this manuscript, the *Necator* specimens, different from *N. americanus* and shared by researchers and gorillas at DSPA, are identified as *N. gorillae*. The possible synonymy of *N. gorillae* with *N. congolensis* (and possibly also with *N. exilidens*) cannot be ruled out, regardless of the fact, that the two latter species were described from chimpanzees. Interestingly, at the time of the original description of *N. congolensis* by Gedoelst [12], the bonobo has not yet been described as a distinct species. It is probable, that at least one of the two “chimpanzees” that were the source of the type-material of *N. congolensis* originated from Busira Region in Congo, which is, in fact, an area inhabited by bonobos.

Nematode identification has traditionally relied on morphometric data and comparison of morphological structures; morphology-based diagnostics of the nominal species should be further extended by molecular data [15]. Taxonomy is currently dealing with an explosion of sequence, genomic, proteomic and other molecular data [16]. However, the fact that the genomic sequences deposited in databases (EMBL, GenBank, etc.) sometimes refer to misidentified or unidentified organisms complicates analyses [17]. Each DNA sequence should ideally be accompanied by comprehensive identification of the specimen [17–19]. However, this is difficult to achieve with no clear taxonomic check on the name given to a sequence and usually no reference material is retained [17]. Moreover, sequences from the majority of nominal nematode taxa are missing [15]. Generally, the holotype which is declared as the ‘name-bearing’ specimen is too valuable to be ground-up for DNA isolation. Furthermore, type-specimens have often been preserved (sometimes for centuries) with fixatives prejudicial to the preservation of nucleic acid. As such, frequently, the types are absent.

The example of *Necator* spp. in primates and humans shows two possible options in resolving this dilemma. We can give up traditional taxonomy and rely on OTUs based on derived sequences. This approach is broadly used for avian malaria parasites [20]. Alternatively, a maximum effort should be made to collect new well-preserved material, which will be identified by experts and subsequently sequenced. The second challenging option is the only way to continue using the traditional taxonomy important for parasites such as hookworms. This will largely depend on the collaboration between laboratories, institutions, field researchers and wildlife veterinarians studying great apes and their parasites.

## Conclusions

DNA sequences of L3 larvae showed plural species of *Necator* in both humans and great apes in the Central African Republic previously. Based on morphological analyses, we identified the adult hookworms recovered from the faeces of two researchers, in which larvae where also included and identified by Hasegawa et al. [6], as *N. gorillae* and *N. americanus*. This is the first report, supported by the morphology of adult worms, of a species of *Necator* other than *N. americanus* in humans. In order to maintain traditional nematode diagnostics we urge collection of new well-preserved adults suitable for both morphological and molecular examination.
